# Complete genome of chromosome encoding *mcr-1.1* in multidrug-resistant *Escherichia coli* EC332 and EC529 isolates from Vietnamese chicken meats

**DOI:** 10.1128/mra.01200-24

**Published:** 2025-05-07

**Authors:** Tatsuya Nakayama, Takahiro Yamaguchi, Michio Jinnai, Doan Tran Nguyen Minh, Oanh Nguyen Hoang, Hien Le Thi, Phong Ngo Thanh, Phuong Hoang Hoai, Phuc Nguyen Do, Chinh Dang Van, Yuko Kumeda, Atsushi Hase

**Affiliations:** 1 Graduate School of Integrated Sciences for Life, Hiroshima University 12803, Higashi-Hiroshima, Japan; 2 Department of Microbiology, Osaka Institute of Public Health 91397 , Osaka, Japan; 3 Department of Microbiology, Kanagawa Prefectural Institute of Public Health 91350, Chigasaki, Japan; 4 Institute of Public Health, Ho Chi Minh City, Vietnam; 5 Research Center of Microorganism Control, Osaka Metropolitan University 12936, Osaka, Japan; University of Maryland School of Medicine, Baltimore, Maryland, USA

**Keywords:** colistin-resistant *Escherichia coli*, *mcr-1.1*, chromosome, chicken meat, Vietnam

## Abstract

*Escherichia coli* strains EC332 and EC529 were isolated from chicken meat in Vietnam. The chromosomes and plasmids from both strains were sequenced using Oxford Nanopore and Illumina sequencing. The total genome sizes were approximately 5.6 and 5.1 Mbp, respectively, and chromosomes encoding *mcr-1.1* were detected in both strains.

## ANNOUNCEMENT

As antibiotic-resistant bacteria (ARB) have reportedly been found in Vietnamese foods, the transfer of plasmid-derived antibiotic-resistance genes to chromosomes, resulting in antibiotic resistance in ARB, should be of concern. Here, we report the whole-genome sequence (WGS) of *Escherichia coli* harboring a chromosome encoding *mcr-1.1* isolated from chicken meat purchased in Ho Chi Minh City, Vietnam.

**Fig 1:**
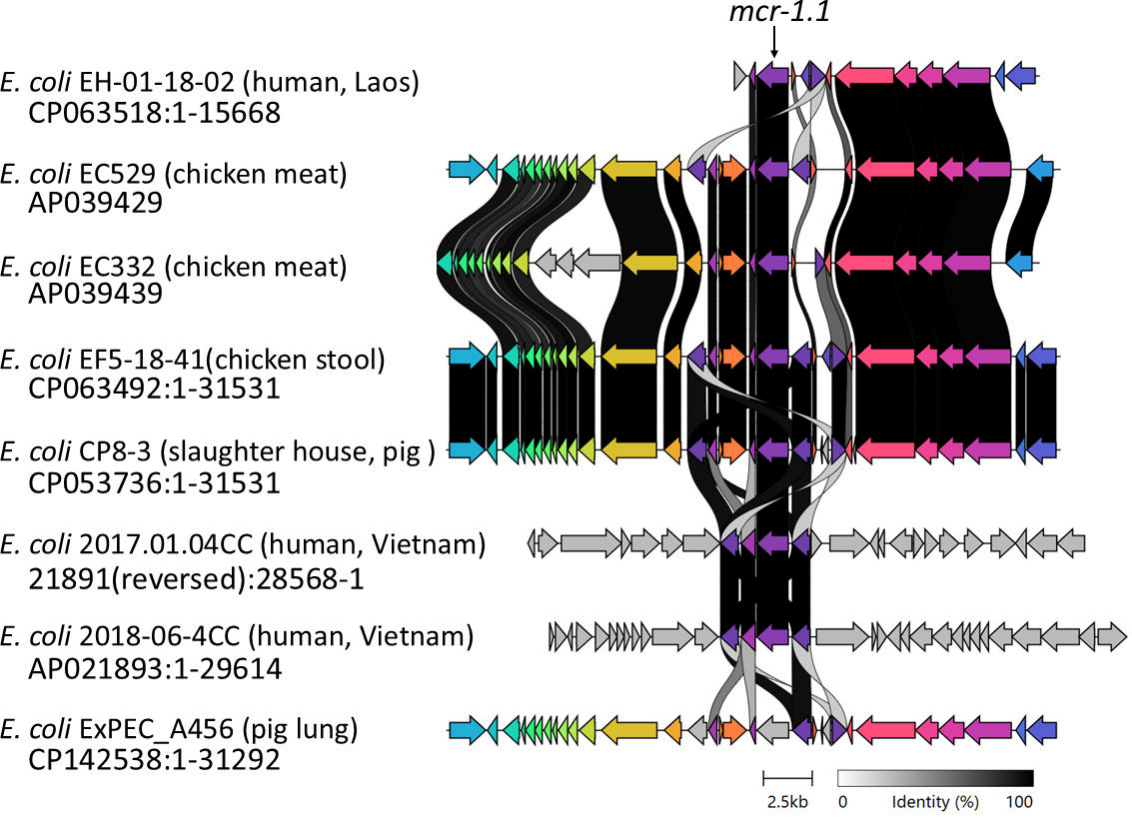
Comparative analysis of *mcr-1.1* encoded on the chromosome in *E. coli* from different sources. A highly homologous genome of the *mcr-1.1* coding region of approximately 2,500 bp was obtained by BLAST. The figure was designed by clinker.

Chicken meat was purchased from a retail market in Ho Chi Minh City. A total of 25 g of chicken meat was mixed with 225 mL of buffered peptone water (composition [g/L]; peptone, 10.0; sodium chloride, 5.0; potassium dihydrogen phosphate, 1.5; and di-sodium hydrogen phosphate dodecahydrate, 9.0) (Merck, Darmstadt, Germany). After incubation at 37°C for 24 h, 100 µL of the culture was spread on CHROMagar COLAPS (CHROMagar, Paris, France) and incubated at 37°C for 24 h. Several mauve, round colonies were selected and isolated. *E. coli* EC332 and EC529, respectively, were investigated for antibiotic susceptibility (1). After subculturing in LB medium at 37^o^C for 18 h, DNA was extracted using the DNeasy Blood and Tissue Kit (Qiagen, Hilden, Germany) and NucleoBond HMW DNA Kit (Takara bio, Shiga, Japan) for short- and long-read sequencing. The extracted DNA was checked using a Qubit dsDNA HS Assay Kit (Thermo Fisher, Waltham, MA, USA). For short- and long-read sequencing library preparation, QIAseq FX DNA library UDI Kit (Qiagen) and Rapid Barcoding Kit (Oxford Nanopore Technologies, Oxford, UK) were used, and sequencing was performed using Illumina Novaseq 6000 (Illumina, San Diego, CA, USA) with a 2 × 150 bp paired-end protocol and MinION (Oxford) with flow cell R10.4.1 (Oxford). After obtaining short-read sequences, trimming and quality checks were conducted using fastp version 0.20.0 (2). Short- and long-read quality checks were performed with FastQC version 0.11.9 (2) and Nanofilt version 2.8.0 (3). Dorado version 0.7.0 was used as the base caller. Hybrid assembly of Illumina (for *E. coli* EC332 and EC529 [total reads, 5,624,224 and 5,096,812; mean length after filtering, 2 × 145 bp reads [paired end]; total bases, 1,577 and 1,548.8 Mb) and MinION (total reads, 64,934 and 147,585; *N*_50_ value, 18,708 and 21,777 bp; total bases, 579.4 and 1,280.5 Mb) sequencing data was performed using Unicycler version 0.5.0 with which circularity was determined (4). Default parameters were used except where otherwise noted. Annotation was performed using DFAST version 1.2.0 software. The assembled WGS was analyzed with MLST2.0 version 2.0.9 and MobileElementFinder version 1.0.2. SpeciesFinder 2.0 confirmed that *E. coli* were isolated, and ResFinder version 4.6.0 analysis showed *mcr-1.1* was encoded in both *E. coli* EC332 and EC529. Clinker version 0.0.29 software was used for comparative analysis of *mcr* (5) (Fig. 1).

Comparisons by basic local alignment search tool (BLASTN 2.16.1+) and clinker image generator showed that the *mcr-1.1* encoded on the chromosome is homologous to the slaughterhouse-derived strain and not to the Vietnamese human-derived strain in the upstream and downstream regions of the *mcr-1.1* (Fig. 1).

**TABLE 1:** Complete genomic information on *Escherichia coli* EC332 and EC529

*Escherichia coli* EC332					*Escherichia coli* EC529				
		Accession number	Length (bp)	GC (%)			Accession number	Length (bp)	GC (%)
Complete genome		AP039429	5,354,426	50.4	Complete genome		AP039439	4,841,278	50.9
Plasmid	pEC332_1	AP039430	113,772	46.2	Plasmid	pEC529_1	AP039440	123,644	51
	pEC332_2	AP039431	72,273	50.5		pEC529_2	AP039441	114,293	49.4
	pEC332_3	AP039432	60,087	52.6		pEC529_3	AP039442	8,086	42.1
	pEC332_4	AP039433	6,477	61.2		pEC529_4	AP039443	3,372	55.1
	pEC332_5	AP039434	5,714	49.6		pEC529_5	AP039444	3,131	45.6
	pEC332_6	AP039435	4,234	55.4		pEC529_6	AP039445	1,549	51
	pEC332_7	AP039436	3,083	39		pEC529_7	AP039446	1,459	50.9
	pEC332_8	AP039437	2,177	46					
	pEC332_9	AP039438	1,981	57.2					

## Data Availability

*E. coli* EC332 and EC529 WGS were deposited in DDBJ/GenBank (accession numbers AP039429 and AP039439) (Table 1). The raw reads were deposited under accession numbers DRX589734, DRX589735, DRX589736, and DRX589737, with BioSamples SAMD00819697 and SAMD00819698, and BioProject PRJDB11927.
